# Predictive Factors for Successful High Myopia Treatment Using High-Frequency Laser-*In-Situ* Keratomileusis

**DOI:** 10.2174/1874364101812010214

**Published:** 2018-07-23

**Authors:** Widya Artini, Setyo B. Riyanto, Johan A. Hutauruk, Tjahjono D. Gondhowiardjo, Aria Kekalih

**Affiliations:** 1 Department of Ophthalmology, Faculty of Medicine, Universitas Indonesia, Cipto Mangunkusumo Kirana Hospital, Jakarta, Indonesia; 2 Jakarta Eye Center Menteng Jakarta, Jakarta, Indonesia; 3 Community Medicine Department, Faculty of Medicine, Universitas Indonesia, Jakarta, Indonesia

**Keywords:** Laser-*in-situ* keratomileusis, Femtosecond laser, Excimer laser, High myopia, Astigmatism, Predictive factors

## Abstract

**Aim:**

To evaluate the predictive factors of LASIK procedure for high myopia with or without astigmatism using a combination of high-frequency femtosecond-assisted LASIK followed by an excimer laser.

**Methods:**

This study was a retrospective interventional case series study to evaluate myopic eyes undergoing high platform LASIK with FEMTO LDV Z2 intervention, followed by WaveLight®EX500 excimer laser machine. Subjects were divided into 2 groups: high myopia (SE of -6.01 to -9.00 D) and very high myopia (SE of -9.01 D or higher). Myopic eyes (Spherical Equivalent/SE) less than –13 D were included in this study. Visual Acuity (VA) was evaluated 1 day and 60 days after the procedure. Predictive factors, such as age, degree of sphere, degree of astigmatism, keratometric reading and axial length were analyzed to detect any influences affecting the final VA results.

**Results:**

A total of 316 myopia eyes underwent intervention, mean age: 25.3±3.8 years. Target treatment was achieved in 96.1% of patients with high myopia and 69.9% of patients with very high myopia. High degree of sphere and astigmatism constitutes an important factor influencing final VA.

**Conclusion:**

Modern machines provide a more promising efficacy and success of LASIK procedure in high myopia: important predictive factors were a high degree of sphere and astigmatism for achieving the optimal final outcome.

## 1 INTRODUCTION

Currently, advanced development of Laser-*in-situ* Keratomileusis (LASIK) is a state-of-the-art technology in refractive surgery, especially for myopia treatment ≤ -13D and astigmatism ≤ -5.00 D [1-4]. According to many studies, by using the conventional (low pulse repetition rates) LASIK instrument, the achievement of final Uncorrected Visual Acuity (UCVA) was within a range of 58.5%-88%, with some undesired symptoms, such as blurred vision, halo or haze [5-7]. When using higher platform LASIK, the percentage of eye achieving an uncorrected distance visual acuity of 20/20 of the target is increased to 90.1%-100% [1, 8-10]. Several predictive factors have been reported to establish successful myopia LASIK treatment, namely, the severity degree of myopia, degree of astigmatism, axial length, corneal thickness, patient’s age, types of LASIK machines, such as ablation profile, laser spot size, cyclotorsion misalignment and time duration of treatment [11-18].

According to many studies, patients with lower sphere and astigmatism may accomplish a better result than with higher sphere and astigmatism (cylinder). The efficacy and predictability of myopia with or without astigmatism are still under discussion and a study conducted by Tomita *et al*. [8] showed a higher outcome of 94.1% in high myopia and 90.7% in very high myopia within ±1.00 D of the intended correction. By using a much faster multifying spot system, a smaller laser spot with overlapping ablation and a higher repetition rate with the potential to track eye movement, the result was more favorable when compared to the use of lower platform LASIK machine [19-21]. To date, no data is yet available to determine the outcome of LASIK treatment, especially in high and very high myopia cases in Indonesia. This is an important factor that necessitated resetting of our normogram to achieve an optimal final outcome. The aim of our study is to assess the related factors for successful LASIK procedure in terms of visual and refractive errors for myopic eyes ≥ -6.00 D to -13.0 D with or without astigmatism using LDV Z2 femtosecond laser, followed by WaveLight®EX500 excimer laser machine.

## 2 METHODS

The Ethical Committee of JEC Hospital approved this study and all patients gave their consent to the LASIK procedures prior to signing the informed consent forms.

## 2.1 Patients

This study was a retrospective study to evaluate patients with myopia ≥ -6.00D to -13.00D, with or without astigmatism, who underwent combined LASIK procedure using femtosecond laser - LDV Z2 (Ziemer Ophthalmic Systems, Port, Switzerland), followed by multifying spot laser excimer, Alcon WaveLight^®^EX500, for ablation of the exposed corneal stroma after exposure of the corneal flap. The LASIK procedures were performed by three experienced surgeons at the Private Eye Care Hospital, JEC@Menteng in Jakarta, Indonesia between October 2015 and June 2016, using the high-frequency LASIK instruments. All related information, such as age, visual acuity pre-post LASIK, degree of myopia, degree of sphere, degree of astigmatism, axial length, keratometric reading and corneal thickness and outcome at the 2-month follow-up of LASIK procedures were retrieved from medical records.

## 2.2 Inclusion And Exclusion Criteria

The inclusion criteria constituted patients over 18 years and under 35 years of age with myopia of ≥ -6.00D to -13.00D, with or without astigmatism, with no previous history of any disorders, such as dry eye, cornea, lens or retina, ocular trauma or eye surgery, and presenting with stable refraction error over a period of one year. The exclusion criteria were those with systemic diseases (diabetes mellitus, systemic connective tissue disorders (arthritis or lupus) etc.), pregnancy, breastfeeding, keratoconus, residual stromal bed thickness less than 250 µm, intra- or postoperative complications, and those presenting with incomplete data. Before performing corneal topography to detect eye disease, deformities and various other vision-related issues, soft-lenses must be removed 2 weeks prior to intervention and in the case of semi-rigid lenses, the patients should discontinue wearing them at least 3 weeks prior to intervention. Only one eye from each patient (higher myopia) was used for analysis of participation in this study.

## 2.3 Data Collection Pre- and Post-Lasik Treatment

Uncorrected Visual Acuity (UCVA) and Best Corrected Visual Acuity (BCVA) were obtained using the Snellen chart, converted into LogMAR notation and calculated into a Spherical Equivalent (SE): Sphere in diopters + (1/2 cylinder diopters). Corneal thickness measurement and corneal topography were obtained using Pentacam^TM^ (Oculus, Wetzlar, Germany), eye axial length and keratometric reading were measured using the IOL Master (Carl Zeiss AG, Oberkochen, Germany), and slit lamp examination evaluating the anterior and posterior segments (Haag-Streit, BQ, USA) was also performed. A retinal subspecialist examined peripheral retinal assessment for all patients before the procedure.

Subjects were divided into 2 groups according to their degree of myopia: Group 1 (high myopia): SE -6.01 to -9.00 D and Group 2 (very high myopia): SE -9.01 D or higher.

The LASIK procedure was considered successful if the patient achieved BCVA of 6/6 with refractive errors ≤ ±0.50 D. The efficacy measure was the proportion of eyes achieving an uncorrected visual acuity of 20/20. The predictability measure was refraction within ±0.50 Diopters (D) of mean target spherical equivalent refraction. The safety index (postoperative decimal BCVA/preoperative decimal BCVA) and efficacy index (postoperative decimal UCVA/preoperative decimal BCVA) were also calculated. The predictive factors were age, type of myopia, axial length, keratometric reading, corneal thickness, the degree of sphere and astigmatism.

## 2.4 Lasik Technique

All subjects were given sufficient information regarding the LASIK procedure and presented signed informed consent forms prior to intervention. Xylocaine 4.0% ophthalmic solution was administered for a duration of 30-minutes and immediately prior to initiating femtosecond laser procedure.

## 2.5 Femtosecond Laser Photodisruption

A femtosecond laser LDV Z2 (Ziemer Ophthalmic Systems, Port, Switzerland) with a high numerical optic aperture was used in this study to create a lamellar flap with smooth in-plane raster cut for the eyes of all subjects. The femtosecond was optimized with ultrafast pulses frequency (> 5 megaHz pulse repetition rate) with 200-350 fs (100 x 10^-15^) pulse duration ≤2 μm spot size and low laser pulse energy (~ 2 nJ/pulse). Using the principles of photoionization (laser-induced optical cutting profile), photodisruption was introduced to create 90- to 100 μm corneal flap thickness depending on the required residual corneal bed thickness (250 – 300 μm).

Before starting the procedure, one drop of viscoelastic solution was applied to the surface cornea and suction ring placed at the center of cornea. 8-10 seconds were required to perform photodisruption with the side-cut spot and overlapping to create a stable, smooth corneal flap. After a 9.0 mm diameter superior hinge and an inverted side cut angle of 120^0^ programmed corneal flaps were formed, the flaps were flipped back on the hinge and the exposed corneal bed was cleaned with merocel sponge (Alcon, Forth Worth, TX, USA).

## 2.6 Excimer Laser Photoablation

An excimer laser, WaveLight®EX500 (Alcon Laboratories, Erlangen, Germany) was used. This has a Wavefront-optimized and Wavefront-guided treatment profile and is equipped with compromised nomogram. The high-ablation speed laser has specific features that can produce an extremely small beam size overlapping ablation, including full-width-at-half-maximum diameter with super-Gaussian beam profile, Intelligent Thermal Effect Control (ITEC), Automatic Fluence-Level Adjustment (AFLA) with 1.4 seconds per diopter ablation time, and customized pupil-center centration option during light condition with dynamic pupil tracking from 1.8-8.00 mm. To compensate for eye movement during LASIK procedure, the ablation frequency was set at 500 Hz and synchronized at 1050 Hz.

The instrument was checked thoroughly before being put to use for the first case of the day. The optical zone was selected at 6.0 mm for correcting high/very high myopia. Residual corneal stromal bed thickness was determined as approximately 250 μm. During the ablation procedure, the corneal stroma was exposed to laser and the patients were informed to remain still and fix their vision on a target red-green light. The duration of procedure was about 10-18 seconds. After the ablation procedure was completed, all debris was irrigated using balanced salt solution (®BSS) and merocel sponge, the corneal interface cleaned and the corneal flap replaced to its proper position. One drop of both moxifloxacin hydrochloride 0.5% ophthalmic solution (®vigamox, Alcon, Fort Worth, Texas) and combined tobramycin and dexamethasone sodium metasulfobenzoate 0.1% ophthalmic solution (®tobradex, Alcon, Fort Worth, Texas) was administered. All patients were instructed to use steroid and antibiotic eye drip as well as artificial tears 6 times daily for 3 weeks. They were also instructed to visit the eye clinic postoperatively for eye examinations on the 1^st^ day, 1^st^ week and 2^nd^ month after surgery.

## 3 DATA ANALYSIS

Management and analysis of data were performed using SPSS program version 22.0 for Windows. Categorical data (predictability of refraction of +0.50 D) was reported as actual numbers and proportion (percentage). Numerical data was presented as mean and standard deviation for data with normal distribution, and as median, minimum and maximum values for data without normal distribution.

To compare the results of visual acuity between pre- and postoperative LASIK procedure in the 2^nd^ month, a paired t-test was performed for all subjects’ eyes, followed by a Wilcoxon test. A chi-Square test was performed to analyze categorical data appropriate for the test and, in some cases a Fisher test was used.

In order to identify the most influential predictive factors, a bivariate analysis was performed followed by a multivariate analysis. All multilinear analyses started with 4 degrees of freedom and applied stepwise regression with the backward removal of the term associated with the highest P value if it were higher than the cutoff of 0.05.

## 4 RESULTS

The total number of 316 eyes participated in our study: Mean age of 25.3±3.8 years, with 40.2% male and 59.8% female participants. LASIK procedure was performed for both eyes in 316 subjects, however, we also included in this study one eye with high myopia found in 217 (64.2%) eyes and very high myopia demonstrated in 99 (35.8%) eyes. The mean corneal thickness was 545.0 ± 29.9 μm, mean axial length 27.1±1.3mm and mean steepening K reading 44.9 ±1.7 D (Table **1**).

### 4.1 Results of LASIK Procedure

Treatment target of SE correction ± 0.50 D in 2 months following the LASIK procedure in all eyes was achieved in 274 (86.7%) eyes. All eyes (100%) in the high myopia subgroup and 92.6% eyes in the very high myopia subgroup were within the postoperative refractive errors of + 1.00 D. Moreover, we found that the mean value of preoperative BCVA was 1.00, while the mean value of postoperative UCVA was 0.00, indicating the efficacy index as 0.00/0.00 = 0.00 in high myopia. In addition, we found that the mean value of postoperative BCVA was 1.0; therefore, the safety index was determined as 1.00.

Fig. (**1**) shows the dots in the diagonal line to represent visual acuity as the end result of treatment and to indicate that the LASIK procedure had indeed achieved the expected goals in terms of attempted target.

In the myopia subgroup, treatment target was achieved in 96.1% eyes with high myopia and 69.9% eyes with very high myopia. Table **2** shows the results of the efficacy of LASIK treatment within the subgroups. The mean preoperative UCVA (logMAR), mean sphere, mean astigmatism and the SE in high myopia and very high myopia subgroups before the LASIK procedure showed improvement after LASIK procedure with a statistically significant difference (*p* < 0.001). No eyes demonstrated an adverse effect (vision loss) following LASIK treatment. BCVA showed significant improvement in high and very high myopia. Á statistical difference was found in high myopia compared to very high myopia in terms of high degree of the sphere and spherical equivalent (Table **2**).

### 4.2 Predictive Factors Associated with Successful Treatment

Include grade of myopia, spherical equivalent (*p* = 0.001), high degree of sphere and astigmatism (*p* = 0.001), LogMAR (*p* = 0.006) and axial length (*p* = 0.001), as shown in bivariate analysis (Table **3**).

In multivariate analysis, the variables revealing predictive risk factors that could possibly affect successful LASIK procedure include the high degree of sphere with an odds ratio of 1.82 and a high degree of astigmatism with an odds ratio of 2.63, as shown in Table **4**.

## 5 DISCUSSION

The aim of our study was to evaluate the early postoperative outcome of LASIK using higher frequency excimer laser platform for the treatment of high degree myopia with or without astigmatism. Excimer laser with high-speed repetition rate laser pulses prevents dehydration of the exposed cornea eye and maintains fixation point during corneal ablation processing aided by a multi-dimensional eye tracker in all four dimensions to compensate eye movements and iris recognition assisted tracking. The high ablation speed laser has specific features to ensure an extremely small beam size and overlapping, also to create a smoother cut that could well affect the ablation validity. By using this high platform excimer instrument, a more promising and predictable result in visual acuity goal can be anticipated, even in those patients with a severe degree of sphere and astigmatism [8, 9, 22-25]. In addition, combined with the up to date technology the femtosecond laser machine is able to create a thinner corneal flap, thus providing a thicker stromal bed that may withstand more ablation, as well as a smoother and more stable corneal flap [26-30].

The outcome of this study supported the assumption that the newer combined LASIK platform with higher repetition rate provides a better success rate compared to the previous studies on high degree myopia that used lower platform LASIK [10, 31-44]. Our study had an efficacy of 86.7% within ±0.50D in all eyes, as shown 2 months after LASIK procedure, and higher efficacy was found in eyes with high myopia (96.1%) followed by very high myopia (69.9%). A study conducted by Tomita *et al*. ^8^ which included patients with myopia or myopic astigmatism with SE of -0.50 to -11.63 D, showed a comparable outcome in high myopia correction using another high platform LASIK machine. The mean preoperative SE in the high myopia subgroup was -7.32 D and -9.85 D in the very high myopia subgroup. The mean postoperative error at the 3-month follow-up was 97.7% eyes with high myopia and 95.3% eyes with very high myopia. This result was within ±1.00 D of the intended target with mean SE of 0.10 D in the high myopia subgroup and -0.07 D in the very high myopia subgroup. Using the intended treatment target of±1.00D, our study showed a better efficacy with 100% eyes in the high myopia subgroup and 92.6% eyes in the very high myopia subgroup with postoperative refractive errors of ±1.00 D. Kanellapoulos AJ *et al*. [10]. using the Alcon-WaveLight Refractive Suite with preoperative UCVA of 0.001 to 0.8 (in decimal), conveyed that in high myopic eyes achieving postoperative refraction spherical equivalent in the -0.50 to 0.00 D was 87% after the 12-month follow-up. The explanation of this finding might be related to discrepancy of measurement and treatment position of the eye due to laser misalignment or cyclotorsion in high myopia, also the compensation of the loss of ablation efficiency when the laser hit the steeper cornea, which might have possibly contributed to the final result. ^10^ Nevertheless, our study clearly confirmed a more optimal achievement in high myopia treatment. When compared to the study by Alio *et al*.^6^ when using micro-keratome combined with A 193 nm VISX 20/20 excimer laser, the efficacy outcome of LASIK was 88% in high degree of myopia (-6.00 to -10.00) in the 3-month follow-up. Furthermore, his report on high myopia treatment states that the outcome of the 15 years observational study confirms that the efficacy regressed to 46.15% within ±1.00 D [44]. While Al-Zeraid *et al*. [43] reported the use of IntraLase femtosecond for correction of moderate to high astigmatism ranging from 2.5 to 4.5 D (mean -3.22 ±0.59 D), a 61% efficacy was determined after the 6-month follow-up. Both study results appeared to indicate a relatively low efficacy outcome when compared to our study. This confirms that higher-frequency LASIK platform machine femtosecond combined with high excimer LASIK may offer excellent results regarding efficacy and predictability for high sphere-astigmatism eyes. The efficacy differences compared to the previous reports strongly relate to the different platform LASIK machines.

It was found necessary to reset our normogram in cases of very high myopia, especially with high astigmatism, in order to achieve a more optimal intended SE. In every LASIK procedure for high myopia, each patient required an adjustment of minus sphere against the normogram data provided by the excimer LASIK instrument [45-47]. The discrepancies in very high myopia, mostly with astigmatism, reflected that a new recalculated normogram needs to be considered to our excimer machine system. Astigmatism vector analysis should be included in our calculated normogram [48, 49].

For individual parameters, it was clearly shown that all eyes with high myopia showed a significant increase in UCVA after LASIK procedure, and SE in these groups was found to have decreased significantly after treatment. These results denote that LASIK procedure was able to reduce the degree of myopia and spectacle or contact lens dependency by providing better UCVA [50, 51]. In terms of BCVA, very high myopia eyes in these myopia groups showed better post-LASIK BCVA compared to pre-LASIK condition with no statistical significance, thus demonstrating a good indication of the outcome. This result implied that LASIK is a safe procedure with no signs of ectasia and hyperopic spherical equivalent ≥ -0.5D [52-56]. The above fact supports our assumption that this procedure may be considered safe when applied to eyes with a high degree of myopia ≤ -13 D.

LASIK procedure for those patients with high degree of myopia, particularly with high astigmatism, still remains a huge challenge. The precise predictability of final visual acuity needs to be further determined by residual refraction. In order to address this matter, factors that can affect the precision of attempted target visual acuity were analyzed by performing bivariate and multivariate analyses to include age, the degree of sphere-astigmatism, axial length, K reading and type of myopia. It showed that degree of astigmatism expressed in diopters was an important predictive factor followed by a degree of sphere. There are also a number of factors, which may lead to less favorable end results. According to the multivariate analysis in our study, eyes with a higher degree of sphere and astigmatism possessed a higher risk of failure with OR 1.82 (95% CI 1.50-2.19) and 2.63 (95% CI 1.84-3.76), respectively. A study by Feltham *et al*. [57] supported this result. They found that refractive errors ≤-5.00 D sphere had a lower risk of failure when compared to the preoperative refractive errors ≥-5.00 DS with OR 0.41 (95% CI 0.2-0.7). This conclusion has also been supported by a number of other studies [58-60].

It is well-known that a high degree of sphere in high myopia is strongly associated with the axial length of the eyes [61-63]. Steeper central cornea with thicker peripheral area is known to alter the biomechanics of cornea, disturb corneal integrity and increase postoperative spherical aberration [64-68]. Moreover, in high refractive error, the necessary large ablation depth is also limited to the restricted amount of residual stromal bed thickness. The residual stromal bed should remain at 250-300 μm in order to avoid the risk of corneal ectasia [69, 70]. However, as the degree of a sphere or high astigmatism increases, while the available stromal bed is still limited, this may result in residual amount of myopia, *i.e.* not be within the intended target, thus affecting the efficacy of LASIK treatment for eyes with very high degree of spherical astigmatism [22, 35, 44, 70-75].

The limitation was retrospective nature of this study design. Some confounding factors cannot be avoided, such as doctor preference, achievement target, stability observation in follow-up or astigmatism vector calculation in normogram setting.

However, we are of the opinion that this study represents a similar efficacy and safety profile when compared to other studies. In addition, our study provides strong and sound evidence that high-frequency femtosecond laser followed by modern excimer laser platform may ensure a promising, predictable, effective, safe and successful LASIK procedure for a high range of myopia.

## CONCLUSION

 Our study conveys the important predictive factors to be a high degree of sphere expressed in the power of diopters and degree of astigmatism for achieving the final outcome.

## Figures and Tables

**Fig. (1):**
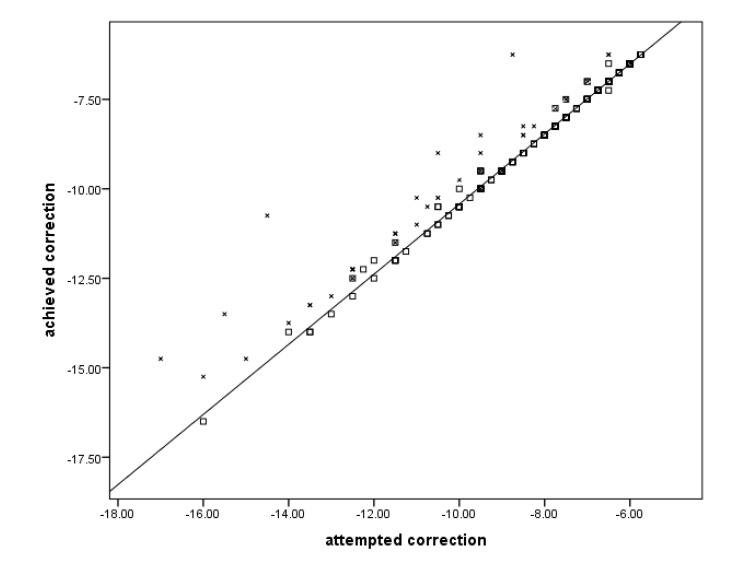
Scatter plot diagram of attempted and achieved correction based on sphere. Subjects within target correction are marked box (□), not within target are marked cross (x). Diagonal line indicates perfect match of attempted and achieved correction.

**Table 1:** Subject characteristics.

Variables	Number (%)
**Gender ^a^**	
Male	127 (40.2%)
Female	189 (59.8%)
Age, years [median (min-max)] ^a^	25.3±3.8
**Degree of myopia ^b^**	
High myopia	217 (64.2%)
Very high	99 (35.8%)
Mean corneal thickness μm (x±SD)^b^	545.0 ± 29.9
Steepening keratometric reading (D)	44.9 ±1.7
Mean axial length, mm (x ± SD)^b^	27.1±1.3
Within target	274 (86.7)

Categorical data was reported as actual number (%)^a^ and numerical data was presented as mean

and Standard Deviation (SD)^b^

**Table 2:** Results of LASIK procedure in subjects with mild, moderate and severe myopia.

**VARIABLES**	**High Myopia**			**Very High Myopia**				***P* value based on Anova two way test for Repeated Measurement**
**Median (min; Max)**	**(n:217 Eyes)**			**(n:99 Eyes)**				
(Range)	PRE LASIK	POST LASIK	*P*-Value	PRE LASIK	POST LASIK	*P*-Value	–	
**UCVA (logMAR)**	1 (1; 1.4)	0 (0; 0.2)	Z=12.9; *p*<0.001	1.3 (1; 1.4)	0 (0; 0.3)	Z=8.7; *P*<0.001	F(1,315)=2.677; *P* = 0.103	
**SPHERE (D)**	-7.2(-8.7;-6.2)	0(-0.7; -0.2)	Z=12.8; *P* <0.001	-10.5(-17.5; -9)	0 (-4.2; 0)	Z=8.6; *P*<0.001	F(1,315)=631.5; *P* <0.001	
**Astigmatism**	-1 (-6; 0)	0(-1.5; 0)	Z=12.4; *P* <0.01	-1.5 (-5.2; 0)	0 (-1.2; -0)	Z=8.4; *P* <0.001	F(1,315)=0.377; *P* = 0.540	
**SE (D)**	-8.(-10.7; -6.2)	0 (-1.2; 0.1)	Z=12.6; *P* <0.001	-11.4(-18.5; -9)	0 (-4.5; 0.6)	Z=8.6; *P* <0.001	F(1,315)=189.9; *P* <0.001	

UCVA: Uncorrected Visual Acuity, BCVA: Best Corrected Visual Acuity, SE: Apherical Equivalent, p: Mann-Whitney test

**Table 3:** Bivariate analysis on predictive factors affecting successful LASIK procedure.

	**Within Target**		***P*-Value**
	**Yes (n=274)**	**No (n=42)**	
**Age^t^ year (mean**±**SD)**	25.3±3.5	25.1±3.7	T(314)=-0.354; *p*=0.724
**Grade of myopia^cs^**			
**High n (%)**	209(96.1%)	8(3.9%)	X^2^(1,N=316)= 43.1; *p*=0.001
**Very High n (%)**	65(69.9%)	34(30.1%)	
**SE pre^mw^ median (range)**	-8.6(-18.5; -6.2)	-12.5(-17.7; -7.2)	U=2282; *p*=0.001
**Astigmatism pre^mw^ median (range)**	-1.2(-5; 0)	-2.4(-6.0; -0.5)	U=3234; *p*=0.001
**SPH pre^mw^ median (range)**	-7.7(-16.5; -6.2)	-11.1(-17.5; -6.5)	U=3110; p=0.001
**LogMAR pre^mw^ median (range)**	1(1; 1.4)	1.3(1; 1.4)	U=6250; *p*=0.006
**Corneal thickness^t^ (mean**±**SD)**	545±31	547(25)	t(314)=0.711; *p*=0.477
**K, [mean+SD] ^t^**	44.8±1.7	45.1±1.5	t(306)=1.122; *p*=0.263
**Axial length^t^ (mean**±**SD)**	26.9±1.1	28.4±1.4	t(314)=7.067; *p*=0.001

cs) Chi square test; mw), Mann Whitney test; t), Independent t test

**Table 4:** Multivariate analysis on predictive factors affecting successful LASIK procedure using binary logistic regression stepwise method.

**Predictor**		**B**	**S.E.**	**Wald**	**df**	**Sig.**	**aOR**	**95% C.I.for aOR**	
								**Lower**	**Upper**
	Sphere	0.596	0.096	38.317	1	<0.001	1.815	1.503	2.192
	Astigmatism	0.967	0.183	27.813	1	<0.001	2.629	1.836	3.766
	LogMar	-3.177	1.227	6.706	1	0.010	0.042	0.004	0.462

a. Variable(s) entered on step 1: SperCategories, MRSEprelk, spherisprelk, astigmaprelk, logmarpreL, axial_length
